# Nosocomial transmission of multidrug-resistant Mycobacterium tuberculosis in Spain.

**DOI:** 10.3201/eid0202.960208

**Published:** 1996

**Authors:** J V Rullán, D Herrera, R Cano, V Moreno, P Godoy, E F Peiró, J Castell, C Ibañez, A Ortega, L S Agudo, F Pozo

**Affiliations:** Instituto de Salud Carlos III, Ministerio de Sanidad y Consumo, Madrid, Spain.


### Nosocomial Transmission of Multidrug-Resistant *Mycobacterium tuberculosis* in Spain

Before 1990, outbreaks of multidrug-resistant tuberculosis (MDRTB) were uncommon (1); since then, more than 10 outbreaks have been reported, all in hospitals and prisons in the eastern United States (2-7). Persons traditionally considered at risk for MDRTB (foreign-born TB patients and those inadequately treated for TB) have not been associated with these outbreaks. Instead, the presence of patients with active TB near immunocompromised patients in HIV-dedicated wards has led to MDRTB-infected HIV patients whose TB cases often go unrecognized. The patients receive inadequate treatment in facilities without effective procedures for isolating acid-fast bacilli; these circumstances favor nosocomial transmission. Health officials in other geographic areas where HIV and TB are major public health threats have been alerted to this emerging problem, and surveillance systems have been designed (8).

Spain has the highest reported incidence rate of AIDS in Europe (143.4 cases per million in 1994) (9). Although in Spain TB is not notifiable at the national level, reported rates in the autonomous community of Madrid for 1994 were among the highest in Europe (33.5/100,000) (10). During a 45-month period starting in September 1991, a number of patients and one health care worker became infected with MDRTB in a 120-bed, infectious disease reference hospital in urban Madrid. In May 1995, the Field Epidemiology Training Program of the Spanish Ministry of Health was invited to assist the Madrid Department of Health and hospital officials in investigating the outbreak. This report describes the findings of the epidemiologic and molecular laboratory investigation and analyzes risk factors associated with the outbreak. The study was designed in three parts: 1) a description of the reported MDRTB cases, including a laboratory investigation of isolates; 2) a case-control study comparing HIV-infected patients who also had MDRTB to HIV-infected patients who did not have MDRTB; and 3) a study of tuberculin conversion among hospital employees.

We reviewed the medical records and laboratory specimen testing results of a series of patients with MDRTB in the HIV-dedicated ward of the hospital for January 1991 through June 1995. Cases were defined as patients with culture-confirmed TB and drug resistance to at least rifampin and isoniazid and with no previous history of inadequate treatment. Demographic and clinical variables were collected to characterize the cluster. Drug susceptibility testing and DNA subtyping analysis were performed with resistant strains available at the time of the study. Drug susceptibility testing was performed by the method of proportions in Middlebrook 7H11 medium distributed in petri plate compartments (reference).

All 48 reported cases of isoniazid and rifampin resistance were among HIV-infected patients hospitalized in the HIV-dedicated ward from September 15, 1991, to May 1995. One patient was an HIV-infected nurse who worked on the ward from 1990 to 1994. The mean age of patients was 34.1; 81.3% were male, and 66.6% were intravenous-drug users (Table 1). Of the 47 (97.9%) who died, the mean interval from diagnosis to death was 77.6 days.

The epidemiologic curve suggests a propagated transmission pattern of MDRTB among HIV ward patients, starting in 1991 and continuing until June 1995 (Figure 1). By the first 6 months of 1995, 65% of *Mycobacterium tuberculosis* strains seen among HIV ward patients were multidrug-resistant.

Table 1. MDRTB patient characteristics

| Variable | Number (n = 48) | % |
|---|---|---|
| Sex | | |
| Male | 39 | 81.3 |
| Female | 9 | 18.7 |
| Mean age | 34.1 | (6.8)[a] |
| HIV status | | |
| Infected | 48 | 100.0 |
| HIV risk group | | |
| Intravenous drug users | 32 | 66.6 |
| Homosexual | 10 | 20.8 |
| Others | 6 | 12.6 |
| Outcome | | |
| Death | 47 | 95.9 |
| Discharge | 1 | 4.1 |
| Mean survival after MDRTB diagnosis (in days) | 77.6 | (107.8)[a] |

[a] Standard deviation.

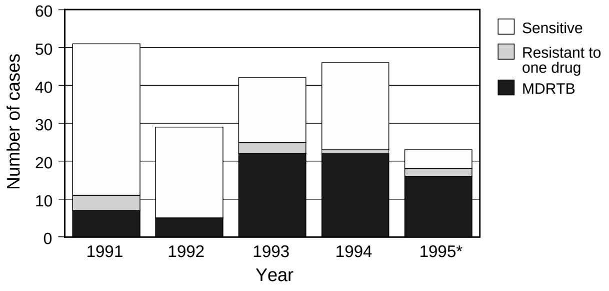

*1995 data are for first 6 months only.

Figure 1. Hospital outbreak, Madrid, Spain, 1995.

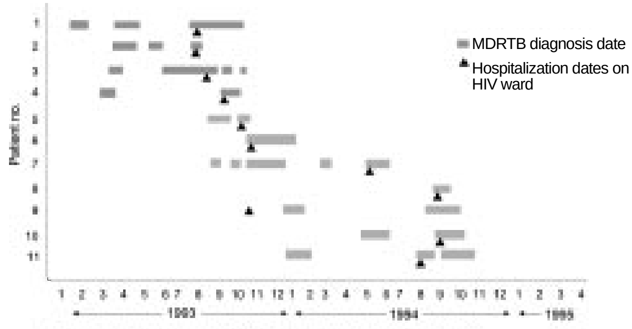

*All strains of resistance pattern HSER (isoniazid, streptomycin, ethambutol, rifampin) had similar DNA subtyping.

Figure 2. Evolution of MDRTB strain* over time, hospital outbreak, Madrid, Spain, 1995.

At the beginning of the outbreak, no consistent antibiogram pattern was observed among isolates. However, beginning in 1993, strains were consistently resistant to isoniazid, streptomycin, ethambutol, and rifampin (HSER); of the 26 patients with an MDRTB diagnosis since the last trimester of 1993, 24 (92.3%) had HSER isolates. DNA subtyping analysis was performed on 12 of these HSER isolates that were available. Eleven of the 12 strains had the same band patterns (Figure 2). Two additional isolates with a different antibiogram pattern had different banding patterns.

Subtyping of *M. tuberculosis* strains was performed (11) by analyzing DNA located between two copies of repetitive sequence IS6110. A 10-µl sample of the extracted DNA was amplified in a reaction mixture containing 0.5 pM of each of the four primers (Ris1, Ris2, Pntb1, and Pntb2), 200 UM DNTPs, 50 mM Tris-HCL, 50 mM KCL (pH 8.8), 2.5 mM MgC12, 0.1% Triton x-100, and 0.5% Taq polymerase. The samples were denatured by incubation at 95$^{o}$C for 10 min and amplified by 30 cycles of denaturation at 94$^{o}$C for 1 min, primer alignment at 56$^{o}$C for 2 min, and primer extension at 72$^{o}$C for 1 min. The amplification products were analyzed by electrophoresis in 2% agarose gel stained with ethidium bromide and observed with an ultraviolet transilluminator.

HIV-infected patients hospitalized on the HIV-dedicated ward between September 15, 1991, and December 31, 1994, who had TB diagnosed in 1994 with a known drug-susceptibility pattern were included in a case-control study. Case patients' isolates were resistant to isoniazid, rifampin, streptomycin, and ethambutol; control patients' isolates were sensitive to the same antimicrobial drugs.

A time-person line diagram was prepared for each of the case patients and control patients, including all hospitalization dates, ward and room number, potentially infectious days, and possible days exposed to infective patients. Prior admission was defined as all admissions to the HIV-dedicated ward since the MDRTB patient was diagnosed (9/15/91) until the end of the period (12/31/94). TB patients were classified as "potentially infective" from the 2 week-period before sputum or culture results were positive for *M. tuberculosis* until the time sputum results were negative or the patient died. TB-negative patients were classified as "possibly exposed" if they were hospitalized on the HIV-dedicated ward during the time a potentially infective patient was also present, beginning on September 15, 1991, until 2 weeks before TB diagnosis.

Thirty-five patients (18 with cases and 17 controls) met the established study case/control definition. Case patients and control patients were not significantly different with respect to age, sex, HIV risk factors, interval between HIV and TB diagnoses or count of CD4 lymphocytes at the time of TB diagnosis (Table 2).

Before the hospitalization during which TB was diagnosed, 76.4% of the case-patients had been hospitalized on the HIV-dedicated ward of the hospital versus 23.5% of control patients (OR = 7.8 [1.4,50.5]) (Table 3). Of the five case patients with no prior hospitalization, three were family members of previously hospitalized HIV-infected patients who had visited the HIV ward frequently during the outbreak period.

Case patients and controls were compared with respect to possible exposure to a potentially infective wardmate beginning on 9/15/91 until 2 weeks prior to the diagnosis of TB. Case patients were more likely to have been exposed to potentially infective wardmates (72.2% with a median of 26.4 days) than control patients (41.2% with a median of 7.6 days). When we stratified possible ward exposure by days (0 days vs. 1 to 30 days vs. 30 days), we observed a dose-response effect, and the chi-square for linear trend was statistically significant (Table 3). Case patients were more likely to die during their initial hospitalization for MDRTB(94.4%) than were control patients (29.4%) during their hospitalization for TB (OR = 40.8 [3.6,1842]) (Table 3).

A TB screening clinic visit was offered to all hospital employees after the outbreak was identified. Of the 591 active employees, 565 (95.6%) participated. Of these, 288 (51%) had not participated in previous hospital employee screening programs conducted in 1990 and 1994. The overall prevalence of TB infection among participating employees was 450 (80%) of 565; only 115 (20%) of the current employees tested were tuberculin-negative.

Employees currently working at the hospital, who had a documented negative (< 6 mm) tuberculin test between January 1993 and June 1995 were eligible for this skin test conversion study. Many of the employees had received BCG vaccine. For those who had not received BCG vaccine, conversion was defined as an induration of 10 mm or greater with a change of at least 6 mm of induration since the last negative tuberculin test (12). For BCG vaccinees, conversion was defined as a 15-mm induration change since the last negative (< 6 mm) skin test.

Employees were defined as occupationally exposed if they worked in parts of the hospital where exposure to patients or *M. tuberculosis* was likely (the HIV-dedicated ward, HIV outpatient clinic, radiology unit, the mycobacteriology laboratory, and the internal medicine ward). Employees were asked to quantify the cumulative number of months spent in these high-risk areas, regardless of their usual place of work, during the 30-month study period.

According to the Mantoux technique, 2 tuberculin units of purified protein derivative (PPD) tuberculin RT-23 were administered in the anterior forearm of the screened employees, and the results were read within 48 to 72 h. Of the participants, 92 (16.3%) were eligible for the conversion study. The incidence of conversion during the 30-month period was 24 of 92 (26%). Employees who had occupational exposure to high-risk areas had higher conversion rates than employees who did not have occupational exposure to high-risk areas (RR = 5.0 [2.7,9.6]) (Table 4). The conversion had a dose-response effect, that is, the more months the person is occupationally exposed to high risk-areas, the higher the risk for conversion.

This is the first nosocomially transmitted MDRTB outbreak reported in

Table 2. Population characteristics: case control study

| Variable | Case patients (%) n = 18 | Control patients (%) n = 17 | OR (95% CI)[a] |
|---|---|---|---|
| Mean age (yr) | 34.4 | 35.2 | $p = 0.30$[b] |
| Male sex | 13 (72.2) | 14 (82.3) | 1.8 (0.3-12.1) |
| IVDU | 11 (61.1) | 11 (64.7) | 1.2 (0.2-6.7) |
| Interval HIV to TB diagnosis in days | 1403 | 1293 | $p = 0.79$[b] |
| CD4 lymphocyte count, median | 112.9 | 199.5 | $p = 0.30$[b] |

[a] OR (95% CI) = odds ratio (95% confidence intervals); Fisher's Exact Test.
[b] *p* value for analysis of variance (ANOVA). IVDU = intravenous drug user.

Table 3. Variables associated with MDRTB

| Variable | Case patients (%) n = 18 | Control patients (%) n = 17 | OR (95% CI)[a] |
|---|---|---|---|
| Prior admission to HIV ward | | | |
| Yes | 13 (76.4) | 5 (29.4) | 6.2 (1.2-37.2) |
| No | 5 (23.6) | 12 (70.6) | |
| Possible ward exposure days | | | |
| None | 5 (27.8) | 10 (58.8) | 1.0 |
| 1-30 | 6 (33.3) | 5 (29.4) | 2.4 |
| 30 | 7 (38.9) | 2 (11.8) | 7.0, $p = 0.03$[b] |
| Outcome | | | |
| Death | 17 (94.4) | 5 (29.4) | 40.8 (3.6-1842) |
| Discharge | 1 (5.6) | 12 (70.6) | |

[a] OR (95% CI) = odds ratio (95% confidence intervals); Fisher's Exact Test.
[b] Chi-square for linear trend.

Table 4. Employee tuberculin screening conversion study results

| Variable | Convertors (%) n = 24 | Nonconvertors (%) n = 68 | RR (95% CI) |
|---|---|---|---|
| Occupational exposure to high-risk areas | | | |
| Yes | 14 (70.0) | 6 (30.0) | 5.0 (2.7, 9.6) |
| No | 10 (13.9) | 62 (86.1) | |
| Months exposed in high-risk areas | | | OR[b] |
| 0 | 6 (10.7) | 50 (89.3) | 1.0 |
| 1-6 | 4 (28.6) | 10 (71.4) | 3.3 |
| 7-36 | 14 (63.6) | 8 (36.4) | 14.6, $p < 0.01$ |

[a] RR (95% CI) relative risk (95% confidence intervals).
[b] Chi-square for linear trend.

Spain, which, with recent outbreaks in the United Kingdom (13) and Italy, is among the first in Europe. Its characteristics are similar to the other reported outbreaks on that it occurred in an HIV-dedicated ward among non-foreign-born patients who had not been treated for TB; it had a high mortality rate within 3 months of onset during which mycobacteria laboratory surveillance recognized similar antibiogram resistant patterns; and identification of MDRTB isolates was followed by DNA subtyping, which confirmed that the same strain was responsible for the outbreak. Risk factors identified include admission to the HIV-dedicated ward and more “possible exposure” days to potentially infective wardmates; additionally, skin test conversion rates among employees were directly related to working in high risk areas of the hospital. The consistency of these findings with those reported in similar outbreaks and the fact that the same isolate was cultured over an extensive period among many different patients on a hospital ward without proper room isolation techniques support the conclusion that nosocomial transmission was the leading cause of the outbreak.

CDC’s recommended guidelines for hospital TB prevention and control (14) were not fully implemented in the hospital. Acid-fast bacilli room isolation techniques were not in place; moreover, no ventilation system was available to provide negative pressure to prevent bacilli from passing from the MDRTB patients’ rooms to the hallway or to provide the six air interchanges per hour recommended for removing bacilli from room air. Surgical masks used in the HIV-dedicated ward during the outbreak as protective masks are not recommended for this purpose because of filtering and facial sealing problems. Observational visits to the HIV-dedicated ward revealed that MDRTB patients without masks were walking, talking, and smoking in the halls and in the lounge next to the unprotected patients, families, and staff.

Complete follow-up of employees is an essential component of a hospital control program (14). Because 80% of the employees in this outbreak were tuberculin-positive, chest radiographs became an important component for disease screening; yearly surveillance of the tuberculin-negative employees will help determine if the interventions in place to prevent future outbreaks have worked in this setting.

Once the case-control study findings were analyzed, a TB control committee was established. All MDRTB patients were placed in a separate area of the hospital. Hospital staff were informed about the outbreak and alerted about future possible cases and the patient management and treatment schemes. The mycobacteria laboratory expanded the antibiogram service to include all second-line antibiotics used by the hospital clinicians. Clinicians have elaborated a treatment flow chart for MDRTB patients. Masks that fulfilled the sealage and filtering criteria (15) were purchased, and mask use was aggressively implemented. An employee health clinic was also instituted, with a prophylaxis and chest X-ray follow-up program for the 410 infected employees, along with a computerized follow-up surveillance system which included all employees graded by occupational risk category. Plans were made to screen tuberculin-negative employees every 3 months to identify those who had recently seroconverted. Family, community members and wardmates of all patients whose MDRTB had been diagnosed within the previous 6 months were notified of their risk and were scheduled for follow-up evaluations. The HIV-dedicated ward will be transformed into an acid-fast bacilli isolation zone, with an exclusive ventilation system that provides 11 individual rooms, each with 12 air interchanges per hour and negative pressure relative to the hallway.

Health officials in Europe need to be updated about this emerging problem, especially in areas of high HIV and TB prevalence. Basic universal TB control and prevention measures (16,17) should be implemented by all general community hospitals in Spain, especially those with HIV-dedicated wards in areas where TB is prevalent. TB surveillance of health care employees is necessary to identify emerging problems as well as to protect employees, patients, and visitors.

**John V. Rullán M.D.,* Dionisio Herrera, M.D.,* Rosa Cano, M.D.,* Victoria Moreno, M.D.,† Pere Godoy, M.D.,* Enrique F. Peiró, M.D.,* Juan Castell, M.D.,* Consuelo Ibañez, M.D.,* Arturo Ortega, M.D.,† Leopoldo Sánchez Agudo, M.D.,† and Francisco Pozo, M.D.‡**

Instituto de Salud Carlos III, Ministerio de Sanidad y Consumo: *Programa de Epidemiología Aplicada de Campo-Centro Nacional de Epidemiología; †Centro Nacional de Investigación Clínica y Medicina Preventiva; ‡Subdirección General de Salud, Madrid, Spain

Drs. Herrera, Peiró, Castell, and Godoy have received a scholarship from the Fondo de Investigación Sanitaria del Instituto de Salud Carlos III during their 2-year epidemiologic training period in the Field Epidemiology Training Program (Programa de Epidemiología Aplicada de Campo).

### Acknowledgments

We thank the staff of the Center for Clinical Investigation and Preventive Medicine of the Instituto de Salud Carlos III for their collaboration in this investigation; in particular, staff from the following departments: Administration, Respiratory Diseases, Infectious Diseases, Nursing, and the Mycobacteriology Laboratory. For the employee tuberculin study, we thank Drs. Rafael Rey, and Dura; Leopoldo Sanchez Agudo; Margarita Muñoz Arcañiz; Consuelo Yubero Higuera; Concepción Gonzalez de la Calle; Lucia Martin Gadea; and Pilar Fuertes Rodrigues for their collaboration.We acknowledge Dr. Jack Crawford and staff from the Division of Bacterial and Mycotic Diseases, National Center for Infectious Diseases, CDC, for their collaboration as reference laboratory for DNA subtyping analysis; Dr. Daniel Fishbein for editing the manuscript; and Dr. Rafael Rey Durán for providing us with the 1990 and 1994 data set of the hospital employees tuberculin test screening. We thank Luis Dorado for his graphics collaboration.

### References

1. Kent JH. The epidemiology of multidrug-resistant tuberculosis in the United States. Med Clin North Am 1993;77:1391-1409.
2. Edlin BR, Tokars JI, Grieco MH, et al. An outbreak of multidrug-resistant tuberculosis among hospitalized patients with the acquired immunodeficiency syndrome. N Engl J Med 1992;326:1514-21.
3. Beck-Sague C, Dooley SW, Hutton MD, et al. Hospital outbreak of multidrug-resistant *Mycobacterium tuberculosis* infections: factors in transmission to staff and HIV-infected patients. JAMA 1992;268:1280-6.
4. Pearson ML, Jereb JA, Frieden TR, et al. Nosocomial transmission of multidrug-resistant *Mycobacterium tuberculosis*: a risk to patients and health care workers. Ann Intern Med 1992;117:191-6.
5. Fischl MA, Uttamchandani RB, Daikos GL, et al. An outbreak of tuberculosis caused by multiple-drug resistant tubercle bacilli among patients with HIV infection. Ann Intern Med 1992;117:177-83.
6. Dooley SW, Villarino ME, Lawrence M, et al. Nosocomial transmission of tuberculosis in a hospital unit for HlV-infected patients. JAMA 1992;267:2632-4.
7. Centers for Disease Control. Nosocomial transmission of multidrug-resistant tuberculosis among HIV-infected persons—Florida and New York, 1988-1991. MMWR 1991;40:585-91.
8. Ausina V, Riutort N, Viñado B, et al. Prospective study of drug-resistant tuberculosis in a Spanish urban population including patients at risk for HIV infection. Eur J Microbiol Infect Dis 1995;14:105-10.
9. WHO-EC Collaborating Centres on AIDS. AIDS Surveillance in Europe-European Centre for the epidemiological monitoring of AIDS. Quarterly Report #45; March 1995.
10. Boletín Epidemiológico de la Comunidad de Madrid 1995. Mayo #5, Vol 4. Informe: Morbilidad por Enfermedades de Declaración Obligatoria, 1994.
11. Friedman CR, Stoecle MY, Johnson WD, Riley LW. Double-repetitive-element PCR method for subtyping *M. tuberculosis* clinical isolates. J Clin Microbiol 1995;33:1383-4.
12. Grupo de Trabajo sobre Tuberculosis. Consenso nacional para el control de la tuberculosis en España. Med Clin (Barc) 1992;98:24-31.
13. Communicable Disease Report. Outbreak of hospital acquired multidrug resistant tuberculosis. United Kingdom PHLS Cornmunicable Disease Surveillance Centre Weekly 1995.
14. Centers for Disease Control and Prevention. Guidelines for preventing the transmission of *Mycobacterium tuberculosis* in health-care facilities, 1994. MMWR 1994;43 (No. RR-13).
15. Lilienfeld, AM, Lilienfeld DE. Foundations of epidemiology. 2nd ed. Oxford, UK: Oxford University Press, 1980;292-5.
16. Maloney SA, Pearson ML, Gordon MT, et al. Efficacy of control measures in preventing nosocomial transmission of multidrug-resistant tuberculosis to patients and health care workers. Ann Intern Med 1995;122:90-5.
17. Wenger PN, Otten J, Breeden A, et al. Control of nosocomial transmission of multidrugresistant *Mycobacterium tuberculosis* among healthcare workers and HIV-infected patients. Lancet 1995;345:235-40.
